# Modeling and Parameter Sensitivity Improvement in ΔE-Effect Magnetic Sensor Based on Mode Localization Effect

**DOI:** 10.3390/mi13050674

**Published:** 2022-04-26

**Authors:** Haoqi Lyu, Zheng Wang, Wuhao Yang, Xingyin Xiong, Zhenxi Liu, Xudong Zou

**Affiliations:** 1 State Key Laboratory of Transducer Technology, Aerospace Information Research Institute, Chinese Academy of Sciences, Beijing 100190, China; lvhaoqi19@mails.ucas.ac.cn (H.L.); yangwh@aircas.ac.cn (W.Y.); xiongxy@aircas.ac.cn (X.X.); liuzhenxi17@mails.ucas.ac.cn (Z.L.); 2 School of Electronic, Electrical and Communication Engineering, University of Chinese Academy of Sciences, Beijing 100049, China; 3 QiLu Aerospace Information Research Institute, Jinan 250101, China; wangzheng02@aircas.ac.cn

**Keywords:** ΔE-effect, magnetoelastic coupling, magnetic field sensing, coupled resonators, mode localization, FEM

## Abstract

A mode-localized ΔE-effect magnetic sensor model is established theoretically and numerically. Based on the designed weakly coupled resonators with multi-layer film structure, it is investigated how the ΔE-effect of the magnetostrictive film under the external magnetic field causes the stiffness perturbation of the coupled resonators to induce the mode localization effect. Using the amplitude ratio (AR) as the output in the mode-localized ΔE-effect magnetic sensor can improve the relative sensitivity by three orders of magnitude compared with the traditional frequency output, which has been verified by simulations based on the finite element method (FEM). In addition, the effects of material properties and geometric dimensions on sensor performance parameters, such as sensitivity, linear range, and static operating point are also analyzed and studied in detail, providing the theoretical basis for the design and optimization of the mode-localized ΔE-effect magnetic sensor in different application scenarios. By reasonably optimizing the key parameters of the weekly coupled resonators, a mode-localized ΔE-effect magnetic sensor with the sensitivity of 18 AR/mT and a linear range of 0.8 mT can be achieved.

## 1. Introduction

In recent years, MEMS resonators have received high attention in high-performance magnetic field sensors [1,2]. Especially in the field of biomedicine, the detection of low-amplitude quasi-static magnetic fields is of great interest, such as real-time biomolecule detection and wearable cardio-encephalographic signal detection [3,4]. It is necessary to develop magnetic field sensors with higher sensitivity, better stability, smaller size, simpler preparation, and lower energy consumption.

Among the mainstream magnetic field sensors, the Hall sensor has a large dynamic range but it is difficult to detect weak magnetic fields below µT with limited sensitivity [5]. Superconducting quantum interferometers (SQUID) can be well qualified for the detection of small-amplitude and low-frequency magnetic fields far below the pico-Tesla range [6], but their large size is difficult to integrate and array on-chip. The tunneling magnetoresistance (TMR) sensors exhibit high sensitivity and lower power consumption [7], but their low-frequency inherent noise, especially the 1/f noise component, is unfavorable [4,8] for the application scenarios where the detection of quasi-static magnetic fields is needed. Therefore, MEMS magnetic field sensors with a high limit of detection (1–100 pT/√Hz) and the larger low-frequency measuring range (0.01–100 Hz) for the weak magnetic fields (below 1 nT) have received much attention [9,10,11,12].

Magnetoelectric sensors based on multilayer thin films have great potential [13,14,15] for the detection of magnetic signals near non-resonant frequencies, especially for quasi-static and low-frequency magnetic fields. Using the direct magnetoelectric effect, the magnetoelectric composite films have high electrical noise caused by parasitic impedance and small magnetoelectric coupling coefficient in nonresonant regimes, resulting in low magnetoelectric sensitivity [9,12,16]. Thus, it seriously affects the performance of the magnetic field sensor.

The above problems can be improved via the frequency modulation principle of the ΔE-effect of thin-film magnetostrictive materials. The ΔE-effect refers to the phenomenon that the elastic modulus of the magnetostrictive material changes with magnetization due to magnetoelastic coupling, which generally occurs in different components of the elastic stiffness tensor [17,18], and the change of the component adds additional magnetostriction strain [19]. The ΔE-effect magnetoelastic sensors are most commonly based on resonant structures, especially cantilevers with a ferromagnetic layer ranging from 100 nm to 2 µm [20,21,22,23,24], which are electrically actuated near the resonant frequency through a piezoelectric layer. For the operating mode among the cantilever-type magnetic sensors utilizing the ΔE-effect, there are the first- and second-order bending modes [22], the torsion mode [18], contour mode [21], and surface acoustic resonators [25] for the magnetic sensitivity improvement. In earlier years, doubly clamped Metglas resonators [26] have a sensitivity of 4.7 Hz/T under a 0.7 mT DC bias. Among the recent developments, a representative nanoplate contour mode sensor [21] obtained a high DC magnetic field sensitivity at 5 Hz/nT. Various ΔE-effect sensors [20,23,24] have shown the ability to detect both low- frequency and amplitude (<1 nT) magnetic fields. In addition, for the sensor model building, in previous works, the ΔE-effect models were only used for magnetic material layer based on the assumption of one-dimensional material deformation [27,28,29] and were not investigated as a magnetic sensor. In recent years, some important works [18,20,22,24,30] have constructed theoretical models of the ΔE-effect magnetic sensors based on the premise of hard-axis in-plane magnetization of paramagnetic materials using quasi-static single-spin approaches, in which the frequency output sensitivity for different working modes is in good agreement with the experiments and explains its magnetic field frequency dependence. However, the sensing mechanism is still limited to the traditional resonant frequency and magnitude output, and the response to the magnetic field is not sufficiently sensitive.

In contrast to the sensors based on the mode localization effect, they exhibit the ultra-high parametric sensitivity (up to three to four orders of magnitude [31,32,33]) and the coupling between the two resonators is constructed mechanically or electrostatically. In the weakly coupled resonator (WCR), a small perturbation will lead to an energy redistribution [34]. In this case, the amplitude ratio (AR) readout metric can lead to ultra-high sensitivity, and other well-recognized advantages of weakly coupled resonant sensors are linearity and immunity to common-mode noise response [33]. The mode localization effect based on the WCR structure has been widely used in inertial sensors and biosensors [35], such as acceleration sensors [36,37,38], and tilt sensors [39], and achieves high sensitivity and low detection limit. Among them, there is a mode-localized magnetometer via the Lorentz force [40,41], the sensitivity of AR is 7800 times higher than that of the frequency, showing the sensitivity of 36.4 AR/T with the resolution of 1.6 μT/√Hz, which unfolds a promising application prospect of the mode localization effect in magnetic field sensors. Therefore, if ΔE-effect sensors can adopt the sensitive mechanism of the mode localization effect, a significant improvement in magnetic sensitivity can be anticipated in such sensors. So far, a numerical and FEM model of the ΔE-effect magnetic sensor using mode localization to predict the sensitivity and the linear range of the sensor is lacking to comprehensively guide the design of this type of sensor.

This paper is organized as follows: The second section first introduces the structure of the coupled resonator. Then, we build a mode-localized ΔE-effect magnetic sensor analytical model theoretically. Based on the designed WCR with multi-layer films, a FEM model is constructed to validate and interpret the analytical model. In Section 3, we investigate the magnetoelastic properties of the WCR and the AR output response under the applied magnetic field. Moreover, the effects of the device material and geometric parameters on the sensitivity and linear range of the sensor are investigated and discussed in detail, which can be used as guidance for the design and optimization of the ΔE-effect magnetic sensor using mode localization effect. Finally, a conclusion of our work is given in Section 4.

## 2. Principles and Methods for Modeling

### 2.1. Sensor Analytical Model

**Figure 1:**
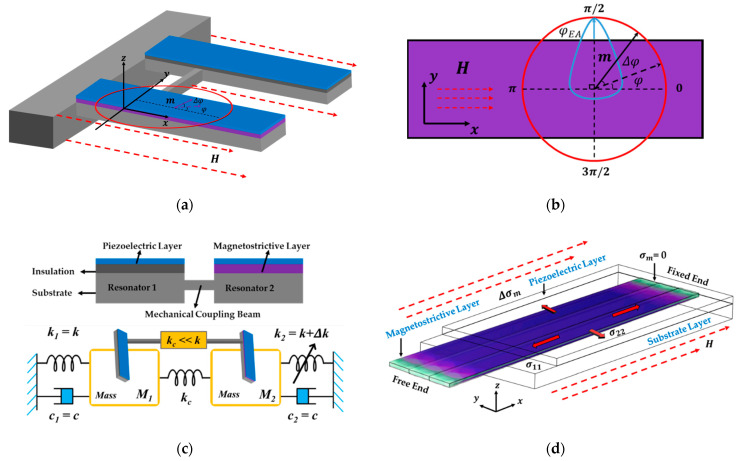
In-plane hard-axis magnetization process of magnetostrictive materials based on the ΔE-effect and the structure of the weakly coupled resonator in our model. (**a**) The 3D structural diagram of the WCR with the change of magnetization and coordinate system used for the sensor analytical model; (**b**) the enlarged top view of the magnetostrictive layer in (**a**). The red circle represents the normalized in-plane magnetization process of the magnetic layer, and the blue area indicates the angular standard deviation of the easy axis; (**c**) top: the cross-sectional view of the WCR, Resonator 2 is sensitive to the magnetic field with a magnetostrictive film, while Resonator 1 is replaced with an insulating layer in the same position. The two resonators are coupled by a mechanical beam; bottom: the equivalent mass-stiffness-damping structure diagram of the WCR, which satisfies the weak coupling condition, where the stiffness of Resonator 2 is regulated by the applied magnetic field; (**d**) the stress distribution of magnetostrictive films under the ΔE-effect is induced after applying an external magnetic field to adjust the resonator stiffness.

The proposed mode-localized magnetic sensor based on the ΔE-effect is realized by the weakly coupled resonator (WCR). For our resonator structure, it specifies the length L  of the cantilever beam, the width w, and the thickness t along the direction *x*, *y*, and *z*-axis, respectively, to establish a space rectangular coordinate system. As shown in the composite films’ stack of Figure 1c, from the top to the bottom, $t_{p}$ is the thickness of the piezoelectric layer, $t_{m}$ is the thickness of the magnetostrictive layer, and $t_{s}$ is the thickness of the substrate polysilicon. The coupled resonator consists of sensitive Resonator 2 (R1 containing a layer of the magnetostrictive film), Resonator 1 (R1 containing an insulation layer of the same thickness), and a mechanically coupled beam to connect R1 and R2.

In our analytical model, we assume the ferromagnetic magnetic particles in the magnetostrictive phase stay in a single domain state, and all of its internal magnetizations are pointed in the same direction. To describe the dependence of the internal uniaxial magnetic anisotropy energy on the direction of spontaneous magnetization, the well-known Stoner–Wohlfarth energy density function based on the spin-orbit coupling theory is used [42], which includes a magnetic crystal anisotropic term, the general Zeeman term, demagnetizing term, and a magnetoelastic term and is given in order by Equation (1):

(1) $$\begin{array}{c} u=K_{u}-K_{u}{\left(m\cdot \theta_{EA}\right)}^{2}-\mu_{0}M_{s}m\cdot H-K_{s}+K_{\sigma }\\ K_{s}=\frac{1}{2}\mu_{0}M_{s}m\cdot H_{d}\;\;,\;K_{\sigma }=-\sigma \cdot \lambda \end{array}$$

where the normalized magnetization vector m and the easy-axis vector $\theta_{EA}$ are denoted by polar angles θ and azimuthal angle *φ* in spherical coordinates. The magnetic vacuum permeability is given by $\mu_{0}$, saturation magnetization by $M_{s}$, effective uniaxial magnetocrystalline anisotropy by $K_{u}$, the shape anisotropy energy density by $K_{s}$, the elastic anisotropy $K_{\sigma }$, and the applied external magnetic field H. The demagnetization field is given by $H_{d}=-M_{s}D\cdot m$, where D is the demagnetization matrix whose effective factors can be estimated by certain geometrical shapes when the internal magnetic field is uniform [43,44]. The total effective anisotropy energy $K_{eff}$ of the magnetostrictive film is expressed by the sum of the three energy density parts in Equation (1), namely $K_{eff}=K_{u}+K_{s}+K_{\sigma }$. When the magnetization reaches saturation, the anisotropic magnetic field is given by $H_{a}=\frac{2K_{eff}}{\mu_{0}M_{s}}$ [45], which is the equivalent field of the effective anisotropic energy density of the spontaneous polarization.

Assuming the spontaneous polarization points to the easy axis ($\varphi_{EA}=\frac{\pi }{2}$), the model defaults to the <100> crystal-oriented hard-axis in-plane magnetization [18,22], and the magnetic hard axis is selected as the sensitive direction (along the *x*-axis), which requires a large external magnetic field to reach saturation magnetization. The out-of-plane components of the magnetization vector m and the easy-axis vector $\theta_{EA}$ are both zero ($\theta_{m}=\theta_{EA}=0$). The normalized magnetization rotation defined by *φ* under external magnetic field is shown in Figure 1a, and Figure 1b is its top view in the magnetostrictive film layer. The blue area represents the rotational standard deviation ratio $\delta_{EA}$ around the mean easy-axis angle $\varphi_{EA}$.

In the orthorhombic crystal system, the ΔE-effect is represented by the change in the elastic matrix ΔC, whose change adds an additional magnetostriction strain [19]. The elastic matrix C is calculated by $C\left(H,\varphi \right)=S^{-1}\left(H,\varphi \right)$. The linear change of the compliance matrix components is given by Equations (2) and (3):

(2) $$S_{ij}\left(H,\varphi \right)=\frac{\partial \varepsilon_{i}}{\partial \sigma_{j}}=S_{m,ij}+\Delta S_{ij}\left(H,\varphi \right)$$

(3) $$\Delta S_{ij}=\frac{\partial \lambda_{i}}{\partial \sigma_{j}}=-\frac{\partial \lambda_{i}}{\partial \varphi }\frac{\partial^{2}u}{\partial \varphi \partial \sigma_{j}}/\frac{\partial^{2}u}{\partial \varphi^{2}}-\frac{\partial \lambda_{i}}{\partial \theta }\frac{\partial^{2}u}{\partial \theta \partial \sigma_{j}}/\frac{\partial^{2}u}{\partial \theta^{2}}$$

The magnetization-dependent part $\Delta S_{ij}$ can be obtained from the equilibrium conditions [18] that are given by the first-order derivatives of 𝑢: $\frac{\partial u}{\partial \varphi }=0,\frac{\partial u}{\partial \theta }=0$. Because of the nonlinear and anisotropic saturation properties of the magnetization of paramagnetic magnetostrictive materials [29,46], the constitutive model for the ΔE-effect nonlinear magnetization function is given by the Langevin function which is based on Boltzmann statistics with a clear physical background [27], given by Equation (4):

(4) $$M_{m}=M_{s}L\left(\left|\psi \right|\right)\frac{H_{eff}}{\left|H_{eff}\right|}\;\;\;\;\;with\;\;\;\;\;\left|\psi \right|=\frac{3\chi_{m}\left|H_{eff}\right|}{M_{s}}$$

Here, $\chi_{m}$ is the initial magnetic susceptibility. The relaxation factor ψ is related to the change of the effective magnetic field $H_{eff}$, which is given by Equation (5):

(5) $$H_{eff}=H+H_{d}+H_{a}\theta_{EA}\cdot \left(m\cdot \theta_{EA}\right)$$

In the linear deformation range of the magnetostrictive material, the stress–strain relationship of the material system satisfies Hooke’s law [47]: $\sigma_{m}\left(H\right)=C\left(H,\varphi \right)\;\lambda_{m}\left(H\right)$, where $\lambda_{m}$ is the magnetostrictive strain tensor and $\sigma_{m}$ is the magnetostrictive stress tensor, which are given in Appendix A and the components of $\sigma_{ij},\;C_{ij},\;\;\lambda_{ij}\;$ will be calculated in Section 3.1.

The ΔE-effect is the overall representation of the response of each component of the compliance matrix to magnetization [17]. The Reuss–Hill approximation based on first principles is a useful method to convert the anisotropic single-crystal elastic constants into isotropic polycrystalline elastic moduli [48]. The shear modulus G and bulk modulus B and can be obtained by $C_{ij}\left(H\right)$, and then, the approximate dependence of Young’s modulus with the magnetic field is obtained according to $E_{m}=\frac{9BG}{3B+G}$ [49], where $E_{m}\;$ is the Young’s modulus of the magnetostrictive film and the derivation is given in Appendix A.

When the frequency of H is much lower than the natural frequency of the cantilever, the distribution of magnetostrictive strain along its longitudinal direction is almost uniform [50], and the normal stresses $\sigma_{22}$ and $\sigma_{33}$ on the film are the same in magnitude and opposite in direction due to the symmetry as shown in Figure 1d. For driving a cantilever beam operating in bending vibration mode, the first resonant frequency $f_{r}$ depends on the force $T_{m}$ applied along its longitudinal direction [51,52]. The effects of shear deformation and rotational inertia can be ignored for slender cantilever beam structure, that is, the change of shear stress $\sigma_{ij}$ can be neglected. Therefore, assuming that the magnetostrictive normal stress is uniformly distributed in the cross-sectional area, using the Rayleigh energy method [53], the expression of the natural frequency $f_{r}$ of the resonator with multilayer films under the magnetostrictive stress is given as a function of the externally applied magnetic field [51]:

(6) $$f_{r}=f_{0}\sqrt{1+\gamma_{n}T_{m}\frac{L^{2}}{2\sum_{i}E_{i}I_{i}}\;}\;\;\;\;with\;\;\;\;f_{0}=\frac{\alpha_{n}^{2}}{2\pi L^{2}}\sqrt{\frac{\sum_{i}E_{i}I_{i}}{A(\sum_{i}\rho_{i})}}$$

$T_{m}$ is given by integration of $\sigma_{11}$ on the cross-section area of the magnetostrictive film given by Equation (7):

(7) $$T_{m}=\iint_{0}^{t_{m}}\left(\sigma_{m}\left(H\right)+\sigma_{m,0}\right)dA$$

where $T_{m}$ and $E_{m}$ are both functions of the H. $E_{i}$, $I_{i}$, $\rho_{i}$ correspond to Young’s modulus, the moment of inertia, and material density of the magnetostrictive layer, piezoelectric layer, and substrate layer, respectively, and $\sigma_{m,0}$ is the prestress along the *x*-axis. The stress correction term [54] is defined as $\gamma_{n}=\frac{2}{\alpha_{n}{}^{3}}\tan h\frac{\alpha_{n}}{2}\left(\alpha_{n}\tan h\frac{\alpha_{n}}{2}-2\right)$. For the first-order natural mode of the cantilever beam, the coefficient is $\alpha_{n}=1.8746$. Resonator 2 serving as a sensitive resonator, the change of the stiffness Δk of Resonator 2 in the applied magnetic field can be obtained from Equation (6) by setting $\omega =\frac{k_{0}}{M}\;$.

Since the coupled resonator is driven around the first-order resonance frequency, in-plane and out-of-plane resonance modes are obtained. Consequently, the 2-DOF (2 degrees of freedom of mass motion) mass-spring-damper system is constructed where the magnetic field acts as the source of stiffness perturbation, and the system model is shown in Figure 1c. It consists of the proof mass M (since $t_{m}\ll t_{s}$, supposing $M=M_{1}\approx M_{2}$), mechanical spring stiffness $k_{0}$, and damping coefficient $c=c_{1}=c_{2}$.

The coupled resonator is driven piezoelectrically by $f_{i}\left(t\right),\;i=1,2$. The energy of the two resonators is injected periodically and the WCR works in the resonant mode. Considering that H=0, the system energy initially presents a uniform distribution. In the case of weak coupling, $\left|\Delta k\right|<\left|k_{c}\right|\ll \left|k_{0}\right|$ [31], where $k_{c}$ is the coupling stiffness. When the H is applied, the stiffness of $M_{2}$ changes slightly and the steady energy distribution is broken, which can effectively suppress the propagation of vibration energy, and the mode localization caused by stiffness perturbation occurs.

According to the above analysis of stiffness perturbation and the assumption of weak coupling, the dynamics of the 2-DOF coupled resonant system can be described by two differential equations:

(8) $${\ddot{x}}_{1}+\frac{\omega_{0}}{Q}\dot{x_{1}}+\omega_{0}^{2}\left(1+\kappa \right)x_{1}-\kappa \omega_{0}^{2}x_{2}=\frac{f_{1}\left(t\right)}{M_{1}}$$

(9) $${\ddot{x}}_{2}+\frac{\omega_{0}}{Q}\dot{x_{2}}+\omega_{0}^{2}\left(1+\kappa +\delta \right)x_{2}-\kappa \omega_{0}^{2}x_{1}=\frac{f_{2}\left(t\right)}{M_{2}}$$

where, $\omega_{0}=\sqrt{\frac{k_{0}}{M}}$, $\omega_{ip}$ and $\omega_{op}$ are the in-phase and out-of-phase natural mode eigenfrequencies of the coupled resonators. $\kappa =\frac{k_{c}}{k_{0}}$ is the stiffness coupling scaling factor and $\delta =\frac{\Delta k}{k_{0}}$ is the normalized stiffness perturbation. $x_{1}$ and $x_{2}$ are the displacements of the two masses, respectively. $Q=\frac{M\omega_{0}}{c}\;$ is the quality factor. For a relatively small coupling strength κ, the two modes of the system are close to each other, and mode overlap is prone to occur. To avoid mode aliasing, it is necessary to satisfy the anti-mode aliasing [33,34] condition $\left|\omega_{ip}-\omega_{op}\right|<\omega_{3dB}$. Thus, the sufficient low damping and higher quality factor need to be considered during the WCR design.

According to the equipartition theorem, the Nyquist relation, and Laplace transformation of differential equations of a 2-DoF mode-localized resonant system, the mode localization theoretical transfer function $H_{i}\left(j\omega \right)$ in the frequency domain can be derived, and the mode frequency $\omega_{i}$ is approximately unaffected by damping under the condition of anti-aliasing [34]. According to the force applied piezoelectrically $f_{2}\left(t\right)>f_{1}\left(t\right)=0$ for the WCR driving, it can be equivalent to an AC $f_{2}\left(t\right)$ exerted on the Resonator 2. The eigenfrequency and amplitude ratio (AR) as a function of stiffness perturbation δ can be expressed as:

(10) $$\omega_{i}\approx {\left(\omega_{0}\left(1+\kappa +\frac{1}{2}\delta -\nu \frac{1}{2}\sqrt{\delta^{2}+4\kappa^{2}}\right)\right)}^{\frac{1}{2}}\;with\;\;i=ip,\;\nu =1;\;i=op,\;\nu =-1$$

(11) $$AR_{i}=\left|\frac{H_{2}\left(j\omega_{i}\right)}{H_{1}\left(j\omega_{i}\right)}\right|\approx \left|\frac{1+\kappa +\delta -{\left(\frac{\omega_{i}}{\omega_{0}}\right)}^{2}+j\frac{1}{Q}\frac{\omega_{i}}{\omega_{0}}}{\kappa }\right|\;\;\;i=ip,op$$

When the AR is chosen as the output metric, the κ and Q of the coupled resonator should be reasonably designed to obtain enhanced parametric sensitivity. Operating in the in-phase vibration mode, the AR has a linear operating range to the right of the veering zone [55] under linear stiffness perturbation δ. However, it can be seen from Equation (6) that there is a nonlinear relationship between δ and the magnetostrictive stress that leads to a decrease in the linearity of the AR output, which will be discussed in Section 3.2. So far, the basic model of the mode-localized WCR with composite films under magnetic field disturbance is established, and AR(H) serves as the output metric.

### 2.2. Sensor Finite Element Model

To verify the mode localization effect of the analytical model under the external magnetic field, a FEM model is constructed based on the structure of the WCR, where the entire device is surrounded by an air domain and an infinite element domain is constructed outside the air domain. To fully calculate the multiphysics coupling effect, the distance between the WCR structure and the boundary of the air domain is controlled to be over 3 times the length of the cantilever beam (maximum size of the structure). A sufficiently dense meshing is built for the magnetostrictive and piezoelectric films while the air domain is sparsely divided. The material parameters and geometric parameters are given in Appendix B and the configuration for FEM is as follows.

As for the material selection for multilayers, the polysilicon is selected for the substrate layer, the AlN for the piezoelectric driving layer, and the Fe-based ferromagnetic material for the magnetostrictive film to build cantilever-type resonators. The coupling method is mechanical and a double-ended fixed straight beam or a folding beam is used as the coupling structure. R2 is a sensitive resonator covered with a layer of magnetostrictive film, while R1 is a follower resonator without magnetically sensitive material attached. The initial displacement and velocity fields are set to zero, and fixed boundary constraints are imposed on the end of cantilevers close to the mechanically coupled beam. To be consistent with the magnetization process of the numerical model, the Langevin function is used as the hysteresis-free magnetization function.

As for the piezoelectric driving, the initial electric potential of the coupled resonator is zero. The DC voltage of $V_{1}=V_{0}$ is applied on the R1 terminal, and the voltage $V_{2}=V_{0}+U_{0}\cos\left(\omega_{v}t+\phi_{v}\right)$ is applied on the R2 terminal. The AC magnitude is $U_{0}$, the angular frequency is $\omega_{v}$ and the phase angle is $\phi_{v}$, and the polysilicon substrate layers in two resonators are both grounded. By coupling the linear piezoelectric constitutive equation with the mechanical motion equation using Equation (12), the coupled resonator is driven to work at its resonant frequency.

(12) $$\sigma_{pi}=C_{p}^{*}\varepsilon_{p}-d_{p}E_{i}\;\;\;\;with\;\;\;\;E=-\nabla V_{i}\;,\;\;i=1,2$$

where $\sigma_{pi}$ is the piezoelectric-induced stress, $C_{p}^{*}=C_{p}\left(1+j\eta_{p}\right)$ is the piezoelectric layer elastic tensor, and $\eta_{p}$ is the damping loss factor. $d_{p}$ is the transpose of electromechanical coupling tensor and $E_{i}$ is the electric field intensity in the piezoelectric layer. Similarly, for the substrate layer, there are $\sigma_{s}=C_{s}^{*}\varepsilon_{s}$ with $C_{p}^{*}=C_{s}\left(1+j\eta_{s}\right)$. Then, the boundary condition of magnetic insulation is applied to the infinite element domain with the zero initial value of the magnetic vector potential, and the uniform background magnetic flux density along the *x*-axis is applied as the sensing magnetic field. The above configuration is solved within the eigenfrequency and frequency-domain study methods in COMSOL Multiphysics^®^ 5.6a and the study results will be compared with the analytical model and fully discussed in Section 3.2.

## 3. Results and Discussion

### 3.1. Magnetostrictive Stress from ΔE-Effect

**Figure 2:**
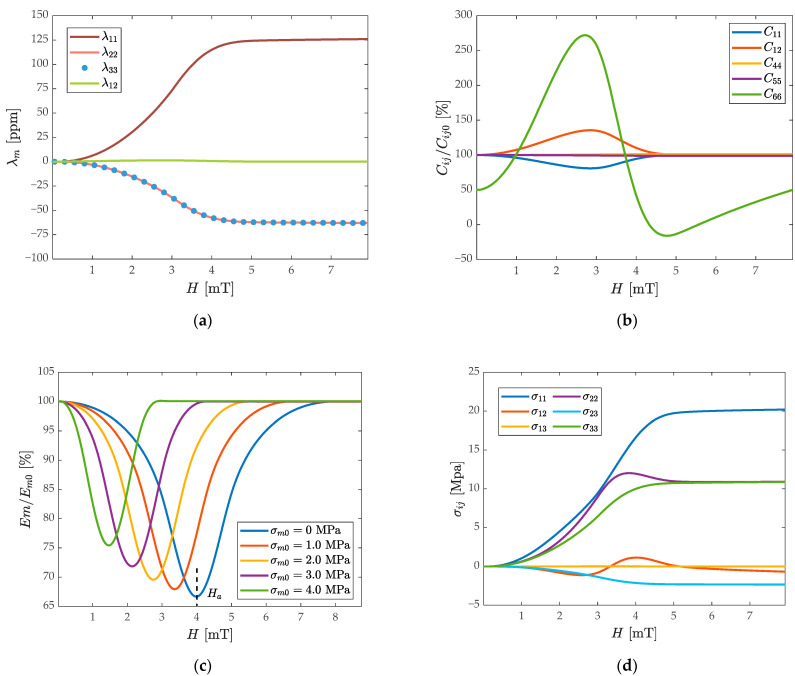
Magnetoelasticity properties of the ΔE-effect: (**a**) the modeled variation of magnetostrictive strain components as functions of the external applied magnetic field H; (**b**) the relative changes of key components of the magnetostrictive material stiffness tensor; (**c**) under the different axial tensile initial stress, the relationship between the normalized Young’s modulus and magnetic field in the magnetostrictive film. When the anisotropic magnetic field $H_{a}$ is reached, $E_{m}$ has a minimum value; (**d**) the dependence of magnetostrictive stress tensor components on the external magnetic field.

With regards to the model result analysis of the ∆E-effect, the initial prestress is set to be zero first. As shown in Figure 2a, based on the in-plane hard-axis magnetization, the magnetostrictive strain tensor components are quantified. With the macroscopic spin magnetization rotating toward the H direction, the magnetostrictive expansion occurs along the *x*-axis, while the compression occurs along the *y* and *z*-axis. With the increase of the applied magnetic field, the component of the magnetostrictive strain tensor increases approximately linearly. Since the out-of-plane polarization is ignored, the shear strains $\lambda_{13}$ and $\lambda_{23}$ are zero and the change rate of the $\lambda_{12}$ is very small, only 1.5 ppm. The normal strain $\lambda_{11}$ has a large variation rate until $H=H_{a}$, and that of $\lambda_{22}$ and $\lambda_{33}$ components are close to half of $\lambda_{11}$. When the $H>H_{a}$, $\lambda_{11}$ increases infinitely close to $\lambda_{s}$. The results in Figure 2b demonstrate the ΔC effect. The changes in the components of the elastic matrix tensor are normalized by their initial value, where $C_{11}$, $C_{12}$, $C_{66}$ are most associated with bending vibrations, and because of the large shape anisotropy of the film, $C_{44}$ and $C_{55}$ remain approximately constant. $C_{11}$ undergoes a process of first softening and then stiffening with the increase of H, reaching a minimum value at $H_{a}$, and returning to $C_{11,0}$ subsequently. The $C_{12}$ change is opposite to the change of $C_{11}$, and $C_{66}$ reaches a minimum at $H_{a}$ and then gradually varies to its initial value. The changes of the above components are continuous, and they are all direct reflections of the positive isotropic magneto-elastic coupling. According to the Reuss–Hill approximation of first principles, the changes of each component are expressed as a whole in the change of Young’s modulus of the magnetostrictive layer, and the ΔE-effect is estimated.

As shown in Figure 2c, Young’s modulus $E_{m}$ of the magnetostrictive layer reaches a minimum value around $H_{a}$, similar to the change of $C_{11}$, which undergoes a process of softening and then hardening back to the initial value. The influence of the initial tensile stress on $E_{m}$ weakens the ΔE-effect shown in Figure 2c; the $\Delta E_{m}$ is reduced and $H_{a}$ also decreases with the increase of the $\sigma_{m,0}$, but the rate of change is almost the same. According to the ΔC effect, the magnetostrictive stress is calculated in Figure 2d. The $\sigma_{11}$ component of the stress tensor increases approximately linearly with the increase of the H. While the normal stress $\sigma_{22}$ and $\sigma_{33}$ are numerically close to half of the $\sigma_{11}$, $\sigma_{12}$ and $\sigma_{13}$ is much smaller than $\sigma_{11}$. For the generation of axial stress, the change in normal stress $\sigma_{11}$ acts as the main source of stiffness perturbation, and the stresses $\sigma_{22}$ and $\sigma_{33}$ due to the geometric symmetry can be canceled. The changing trend of normal stress and strain in this process is in good agreement with the FEM results.

According to Equation (6), the change of $f_{r}$ of R2 is determined by the response of $E_{m}$ on $\sum_{i}E_{i}I_{i}$, $\sigma_{11}$ and $t_{m}$. $\sigma_{11}$ has a stiffness attenuation effect when acting on the cross-section of the integral cantilever beam, which leads to a small change of the resonant frequency and a small stiffness disturbance. As $H_{a}$ is being reached, the total eigenfrequency variation can reach 3470 ppm, which determines the effective range of stiffness perturbations in this WCR system.

### 3.2. Frequency Response and AR Response

**Figure 3:**
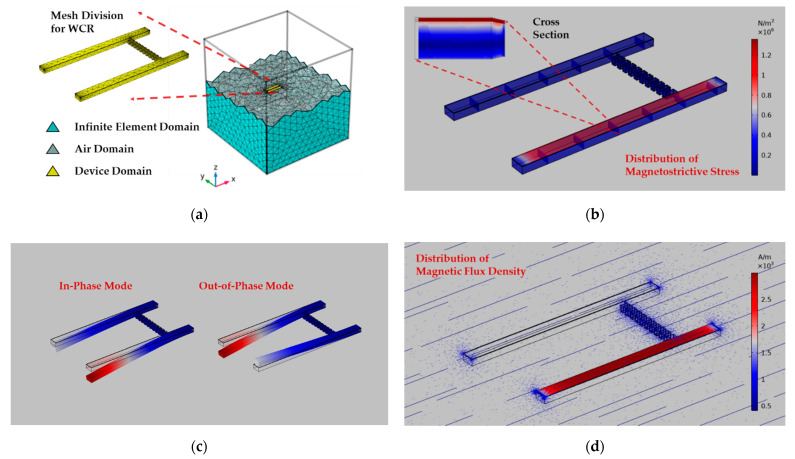
The FEM simulation for the WCR: (**a**) the WCR meshing diagram and the model is divided into an air domain, an infinite element domain, and a device domain (zoom-in); (**b**) stress distribution of the folded-beam mechanically coupled WCR under the 1 mT DC magnetic field along the *x*-axis; (**c**) driving Resonator 2 piezoelectrically, the out-of-phase and the in-phase vibration modes of the WCR; (**d**) magnetic flux density distribution map of the WCR and the convergence within the magnetic film only.

The WCR is driven at the resonant frequency under different values of the H and the coupling structure in this section is a folded beam. The stress distribution, mode shapes, and magnetic flux density distribution are shown in Figure 3. As shown in Figure 3b, when the applied magnetic field is 1 mT, the largest stress is in the magnetostrictive layer. The top left subgraph is an enlarged view of the cross-section along the *x*-axis, which illustrates larger stress is distributed in the central part of the magnetostrictive film along the longitudinal direction with a wide area, and it gradually reduces to zero at both ends, which reflects the rationality of the normal stress $\sigma_{11}$ in the central part as the main source of the stiffness disturbance.

Figure 3d shows the magnetic flux density distribution of the coupled resonator when the applied magnetic field is 4 mT. The magnetic flux density is more concentrated at the corner of the WCR with the edge effects. Neither R1 nor the coupling beam structure has a converging function on the magnetic field. For the sensitive R2, the magnetic flux density in the magnetostrictive film layer is large and uniform and diverges at the fixed constrained end, which shows the magnetization direction under the effective magnetic field $H_{eff}$. The two fundamental vibration modes, $\omega_{ip}$ and $\omega_{op}$, of the coupled resonator are shown in Figure 3c when R2 is driven. The symmetry of the two vibration modes is broken due to different adherent films on each resonator, and the $\omega_{ip}$ mode is used as the sensor working mode. The above results are obtained using Structure 1 whose geometric parameters are given in Appendix B.

**Figure 4:**
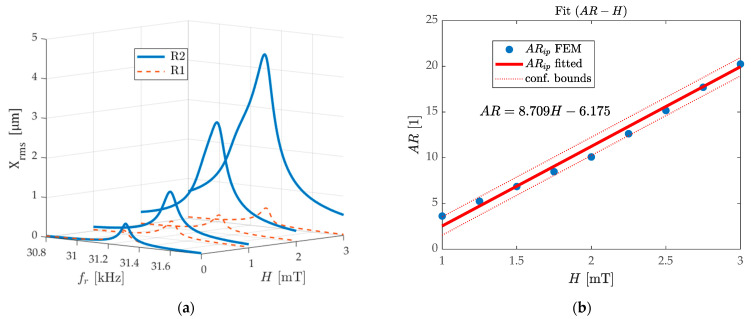

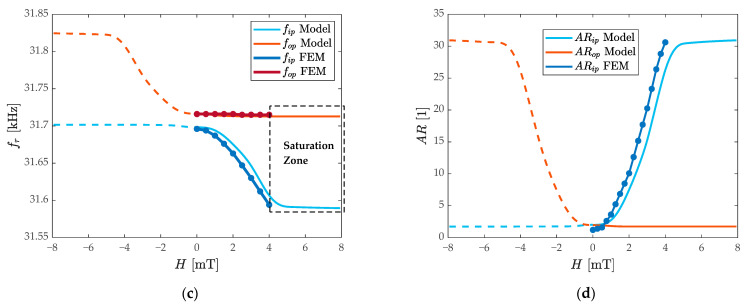
The analytical model and FEM simulation to verify the mode localization effect under the magnetic field disturbance in the WCR: (**a**) the frequency response of R1 and R2 are represented using the displacement RMS magnitude when the applied magnetic field varies from 0 to 3 mT; (**b**) fitting curve of the linear correlation between amplitude ratio and magnetic field with 95% confidence bounds and $R^{2}=0.988$; (**c**) prediction of the eigenfrequency response of the IP and OP modes on the external magnetic field within the comparison of the model prediction and FEM. The dashed box represents the sensor saturation area; (**d**) prediction of the amplitude ratio response of the IP and OP modes on the external magnetic field.

In FEM simulation, in order to obtain a reasonable Q factor and coupling stiffness κ of the WCR for numerical model quantification, the anisotropic loss factor $\eta_{p}=\eta_{s}=0.001$ [56] of the substrate and the piezoelectric layer is applied to simulate the damping term. As shown in Figure 4a, calculated using device Structure 2, the frequency response represented using the displacement RMS (root-mean-square) for R1 and R2 are shown under the applied magnetic field varying from 0 to 3 mT. The blue solid line represents the magnitude of the displacement RMS of R2, and the orange dotted line represents that of R1. With the increase of H**,** the amplitude-frequency response curve of R1 remains almost unchanged, and the displacement amplitude response of R2 gradually increases linearly. Therefore, the resonance peak ratio of the two $\frac{H_{2}\left(j\omega_{ip}\right)}{H_{1}\left(j\omega_{ip}\right)}$ increases nearly linearly. Under the frequency domain verification of FEM, the mode localization effect under the external magnetic field disturbance occurs in this WCR. According to Figure 4a, the quality factor is analyzed when H=0, $Q_{0}=626.29$ is calculated, which corresponds to the typical cantilever-type ΔE-effect sensors and the WCR [20,57,58,59,60]. The coupling factor $\kappa =2.29\times 10^{-4}$ is estimated [61], which meets the anti-aliasing condition $\sqrt{\delta^{2}+\kappa^{2}}\geq \frac{1}{Q}$.

Similarly, using the device Structure 3, the calculation results of its Q and κ are brought into Equations (10) and (11), and the analytical model is used to predict the frequency response and amplitude ratio response of the IP and OP modes. Figure 4c,d are the comparisons of the model prediction and FEM simulation for the eigenfrequency and the AR. The dotted line in the figure represents the negative direction of the magnetic field and the main results are discussed in the case of H>0. Since the stiffness disturbance of R2 has a decreasing trend, when the WCR works in the IP mode, the eigenfrequencies $\omega_{ip}$ decrease, and the OP mode remains unchanged. The FEM results show a 3500-ppm change in $\omega_{ip}$ when $H=H_{a}$ is reached. When $H>H_{a}$, since the magnetization is close to saturation, the model predicts that the frequency reduction rate decays rapidly. The dashed box in Figure 4c represents the sensor saturation area for the eigenfrequencies and the AR output, and the magnitude of $H_{a}$ determines the range of the sensor to some extent, which will be discussed in detail in Section 3.3.

The initial value of the AR above 1 in Figure 4d comes from the asymmetry of the films’ structure of R1 and R2, which should also be from the asymmetry of meshing generation in FEM. The AR sensitivity is predicted by taking the variation range 1~3 mT of the applied magnetic field, and the fitting result by the least-square method with 95% confidence bounds, and $R^{2}=0.988\;$ has a sensitivity of 8.7 AR/mT as shown in Figure 4b. The trend of the model analyses is almost consistent with the simulation results of the FEM simulation. Both of them show an 1850 times improvement in relative sensitivity compared to the eigenfrequency response to magnetic fields using the amplitude ratio output.

### 3.3. Multi-Parameter Optimization Analysis

There are many factors from the material properties of the magnetostrictive film and the geometry of the WCR that affect the sensitivity of the mode-localized amplitude ratio output under the external magnetic field. The specific effects of each parameter on the sensor performance are discussed in this section.

**Figure 5:**
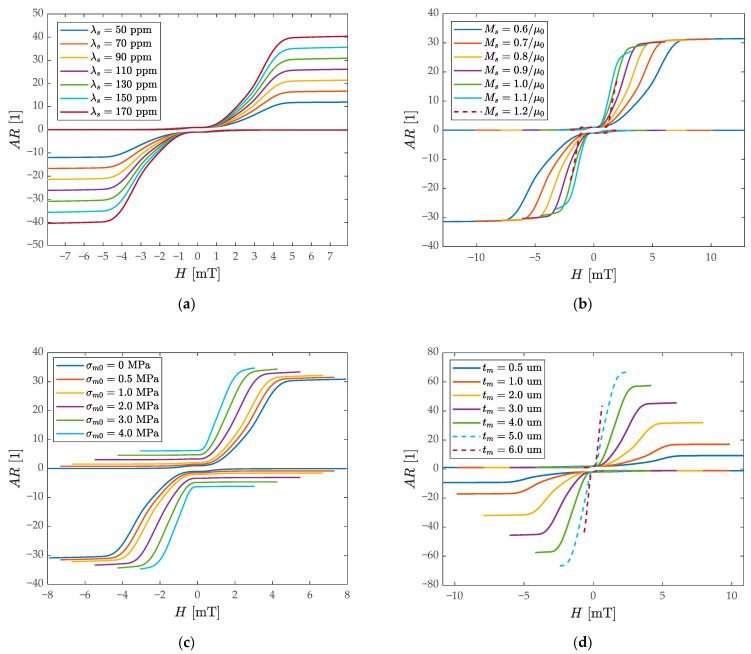
Influence of key materials and geometric parameters on the AR magnetic output curves: (**a**) the response to the AR when changing the saturation magnetostriction $\lambda_{s}$; (**b**) the response to the AR when changing the saturation magnetization $M_{s}$; (**c**) the response to the AR when changing the film tensile prestress $\sigma_{m0}$; (**d**) the response to the AR when changing the magnetostrictive film thickness $t_{m}\;$.

The composite film’s material parameters and device geometry changes are meaningful and achievable based on currently available published works [56,62,63,64]. As shown in Figure 5a, the relationship between the AR and magnetic field is calculated when the saturation magnetostriction $\lambda_{s}$ changes from 50 ppm to 170 ppm. By increasing the $\lambda_{s}$, the slope of the AR curve increases regularly, and the anisotropy magnetic field $H_{a}$ remains unchanged. The red arrow represents the direction of change of the AR curve. The increase in the AR sensitivity is mainly due to the stronger stiffness perturbation caused by the larger magnetostrictive strain according to Equation (A3). As shown in Figure 5b, increasing the saturation magnetization $M_{s}$ can increase the slope of the AR curve in the local region, but at the same time, the caused decrease of $H_{a}$ reduces the linear range of the sensor such as the dark red-dotted curve with large $M_{s}$ in Figure 5b. The improvement of its sensitivity comes from stronger magnetization, and the decrease of $H_{a}$ comes from the restrictive relationship between the $H_{a}$ and $M_{s}$ ($H_{a}=\frac{2K_{eff}}{\mu_{0}M_{s}}$) under the circumstance of a constant $K_{eff}$. Additionally, increasing $M_{s}$ also makes the shape anisotropy weaker in the magnetostrictive film, resulting in attenuation in $K_{eff}$, which reduces the linear range of the sensor by decreasing $H_{a}$.

During the growth of multi-layer films, it is inevitable to bring residual stress in the process, resulting in the WCR having the tensile prestress of the material layer [65]. The model is considered as shown in Figure 5c. When the initial prestress $\sigma_{m0}$ gradually increases from 0 to 4 MPa, the elastic anisotropy energy density $K_{\sigma }$ increases, making $K_{eff}$ decrease and reduces the $H_{a}$ consequently. At the same time, the prestress brings a bias to the decrease of the R2 stiffness, which increases the initial value of the AR, and the AR sensitivity of the sensor remains almost unchanged under the influence of increasing prestress. As shown in Figure 5d, with the increase in the thickness $t_{m}$ of the magnetostrictive film, the $H_{a}$ obviously decreases, while the AR sensitivity increases, which is due to the larger magnetostrictive stress caused by the thicker film. The larger thickness makes the shape anisotropy weaken sharply and the demagnetization factors are no longer equivalent to the thin-film model, which changes the spatial distribution of the internal stray field, and the magnetocrystalline anisotropy is overcome in most regions. The $K_{eff}$ becomes very small and narrow the linear range consequently. (The dashed curve corresponds to a very small linear range and $H_{a}$.)

To fully study the influence of important materials and geometric parameters on the AR sensitivity, the linear range is also an important sensing metric to be considered. Under the variation of different model parameters, the results are based on the magnetic magnitude response range of the nonlinearity less than 1% as the standard for the sensitivity quantification.

**Figure 6:**
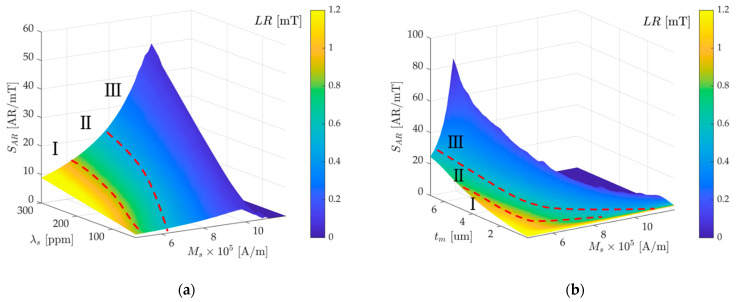
The dependence of the AR sensitivity and linear range for the mode-localized magnetic field sensor based on the ∆E-effect demonstrated by a four-dimensional colormap. Red dotted lines are used to partition zones I–III. (**a**) The response of the curved surface to saturation magnetostriction $\lambda_{s}$ and saturation magnetization $M_{s}$; (**b**) the response of the curved surface to the magnetostrictive thickness $t_{m}$ and saturation magnetization $M_{s}$.

As shown in Figure 6a, by changing $\lambda_{s}$ from 30 to 300 ppm, and $M_{s}$ from $0.6/\mu_{0}$ to $1.5/\mu_{0}$, the relationship between the AR sensitivity and range of measurement is obtained, which is given by a four-dimensional color map (AR sensitivity and range tradeoff surface), and the height of the surface represents the values of the AR sensitivity of the WCR and the shade of color indicates the magnitude of the linear range (LR). From the contour of the surface in Figure 6a, it can be seen that a larger $\lambda_{s}$ and $M_{s}$ bring greater AR sensitivity, but much larger $M_{s}$ will lead to a reduction in the linear range. Therefore, increasing $\lambda_{s}$ is an optimal choice to improve the sensitivity without sacrificing the linear range. The reason for these changes has been explained in the previous section. This surface represents the intrinsic dependence of the sensitivity and LR for mode-localized magnetic field sensors based on the ∆E-effect. For selecting material parameters of the magnetostrictive film, the tradeoff surface is partitioned to zones I–III to be a reference. When considering the design of a large linear range magnetic field sensor, the I-zone is a suitable choice; while in the design of a high sensitivity sensor, the III-zone is more appropriate. The II-zone is a compromising area to be selected, and the parameters need to be selected according to the specific application scenarios.

Since both $t_{m}$ and $M_{s}$ will lead to the change of shape anisotropy and the change of $H_{a}$, the two will cause a superposition decrease effect on the linear range, as shown in Figure 6b, by changing $t_{m}$ from 0.1 μm to 7 μm, and $M_{s}$ from $0.6/\mu_{0}$ to $1.5/\mu_{0}$, the sensitivity and range tradeoff surface is divided into three zones in the same way in Figure 6a. Since excessively large $t_{m}$ and $M_{s}$ make the mode localization effect disappear, it is necessary to avoid selecting large $t_{m}$ and $M_{s}$ at the same time as much as possible.

**Figure 7:**
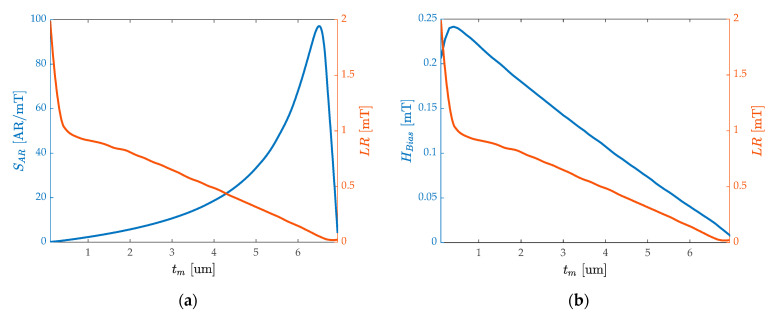
Selecting the projection surface of the saturation magnetization $M_{s}=0.75/\mu_{0}$ from Figure 6b: (**a**) the dependence of the AR sensitivity and linear range on the magnetostrictive film thickness $t_{m}$; (**b**) the dependence of the biased magnetic field and the linear range on the $t_{m}$.

To better reveal the trade-off relationship from the surface, taking the projection surface of the saturation magnetization $M_{s}=0.75/\mu_{0}$ in Figure 6b as an example to draw Figure 7a, the intersection point between the sensitivity curve and the range curve can be found in the function of $t_{m}$. On the whole, the high AR sensitivity is accompanied by a small linear range, which is explained in Figure 5d. As the $t_{m}$ increases within a reasonable range, the sensitivity increases by nearly two orders of magnitude, and the linear range decrease by nearly one order of magnitude. At the intersection of the two curves, the sensitivity is close to 20 AR/mT, and the linear range is about 0.4 mT. Since determining the linear range needs to meet the condition of less than 1% nonlinearity, it is essential to increase the bias magnetic field to change the static operating point. At the same time, $H_{Bias}$ meets the demands of particular application scenarios that require a certain bias magnetic field [66]. As shown in Figure 7b, increasing the film thickness can reduce the initial $H_{Bias}$, which has a similar trend with the curve of the linear range. $H_{Bias}$ determines the static operating point of the sensor and, under the design of high sensitivity, $H_{Bias}$ needs to be taken into account. For example, to achieve a linear range close to the 0.5 mT and 20 AR/mT sensitivity, $H_{Bias}=0.075$ mT is required and $t_{m}=3.5$ μm needs to be deposited.

**Figure 8:**
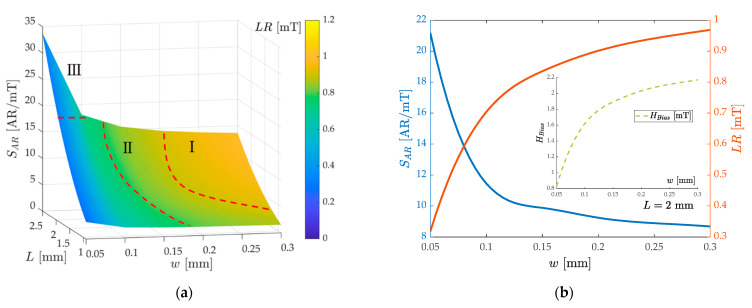

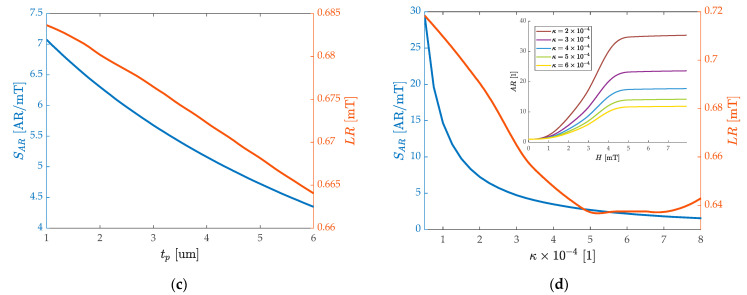
The dependence of the AR sensitivity and linear range with key resonator parameters: (**a**) the response of the AR sensitivity and linear range to the WCR cantilever length L and width w. Red dotted lines are used to partition zones I–III; (**b**) the dependence of the AR sensitivity and linear range on the cantilever width w and the subgraph contains the relationship with the bias magnetic field; (**c**) the dependence of the AR sensitivity and linear range on the piezoelectric film thickness $t_{p}$; (**d**) the dependence of the AR sensitivity and linear range on the coupling stiffness coefficient κ and the subgraph includes the AR magnetic output curves under the different κ.

In terms of the area dimensions of the WCR, as shown in Figure 8a, selecting $t_{m}=2.0\;$μm, $M_{s}=0.8/\mu_{0}$, $\lambda_{s}=130\;$ ppm, the response of the length and width of the cantilever beam to the AR sensitivity and LR is studied. The surface can illustrate obviously that the larger cantilever beam length L and the smaller width w can bring higher AR sensitivity. Consistent with the method of dividing the surface in Figure 6a, it is partitioned into three representative zones I–III for reference. I-zone, II-zone, and III-zone are the large linear range selection, the compromising performance selection, and the high sensitivity selection respectively for the sensor design. Figure 8b reflects the influence of the width of the cantilever beam on the sensitivity and LR when it determines L=2.0  mm. The two sets of curves go in opposite trends and can be selected within a range of sensitivity from 8 to 21 AR/mT and a linear range from 1 to 0.3 mT. It is worth mentioning that the subgraph shows that with the increase in the width of the cantilever beam, $H_{Bias}$ needs to be increased (more than 2 mT $H_{Bias}$ to reach the linear range of 1 mT), which is suitable for the detection with a large bias background field.

When selecting the thickness of the cantilever, only the thickness of the piezoelectric layer $t_{p}$ can be changed due to the fixed substrate thickness. Figure 8c shows that increasing the $t_{p}$ will not only lower the AR sensitivity but also decline the sensor linear range slightly. So, under the premise of piezoelectric driving, the thickness of the piezoelectric layer should be reduced as much as possible to achieve a higher thickness ratio of $\frac{t_{m}\;}{t_{p}\;}$. However, when considering the sensitivity of voltage as output based on the magnetoelectric coupling, for higher magnetoelectric conversion efficiency, the ratio of magnetostrictive and piezoelectric film thickness needs to be adjusted appropriately [67], and the magnetoelectric conversion coefficient under mode localization requires further research. It is well known that reducing the coupling stiffness of the WCR can increase the sensitivity of the mode localization effect [68]. As shown in Figure 8d, the subfigure shows the relationship between the AR and the magnetic field with the coupling coefficient κ ranging from $2\times 10^{-4}$ to $6\times 10^{-4}$, which has a similar trend with the increase of $\lambda_{s}$. The same trend of the sensitivity and linear range curve in Figure 8d illustrates that reducing and optimizing the coupling stiffness of the WCR, such as utilizing of the folded coupled beam to weaken the coupling stiffness, can increase the AR sensitivity and linear range at the same time.

In general, the AR sensitivity and linear range are a pair of contradictions and a compromise needs to be considered. A reasonable selection of material parameters and geometric dimensions in the design process can obtain the target sensitivity and linear range. The film material with high saturation magnetostriction or a weaker coupling stiffness can enhance the AR sensitivity without sacrificing the linear range. Representatively, the mode-localized magnetic field sensor based on ∆E-effect can be predicted to achieve a sensitivity of 18 AR/mT and a magnetic linear range up to 0.8 mT with less than 1% nonlinearity, which shall be verified in open-loop and closed-loop testing. The resolution of the mode-localized magnetic field sensor with the AR as output depends not only on the thermal noise of the WCR but also on the configuration of the interface circuit, which will be discussed and analyzed in detail in follow-up works.

## 4. Conclusions

In this paper, an analytical model and FEM simulation of the mode localization magnetic field sensor based on the ΔE-effect are established to realize the high sensitivity detection of low-frequency and small-amplitude magnetic field. The ΔE-effect of magnetostrictive films is investigated through the anisotropic energy density equation and nonlinear magnetization model based on the uniaxial macroscopic spin-plane magnetization condition. Stiffness perturbation from the ΔE-effect breaks the symmetry of the coupled resonators resulting in the mode localization effect between resonators. Using AR as the output in a mode-localized ΔE-effect magnetic sensor can improve the relative sensitivity by 3 orders of magnitude compared to conventional frequency output.

The effects of material properties and key geometric parameters on the sensitivity and linear range (the nonlinearity is less than 1%) of the mode-localization magnetic field sensor are comprehensively analyzed. According to the model, increasing the saturation magnetostriction, reducing the substrate thickness, and weakening the coupling stiffness factor can effectively improve sensitivity without sacrificing linear range. There are trade-offs between the sensitivity, linear range, and bias magnetic field in the design and optimization of the mode-localization magnetic field sensor depending on different application demands. A magnetic field sensor with the sensitivity of 18 AR/mT and the linear range of 0.8 mT can be realized by adjusting the key parameters of the WCR reasonably. The analytical model can provide design guidance for the mode-localization ΔE-effect magnetic field sensor with high sensitivity and large linear range, which is a promising candidate to meet the demands for biomolecule detection and physiological signal detection in the biomedical field. A mode-localized ΔE-effect magnetic field sensor will be fabricated according to this model using the SOI process to demonstrate the improvement of sensitivity in future work.

## Figures and Tables

**Figure A1:**
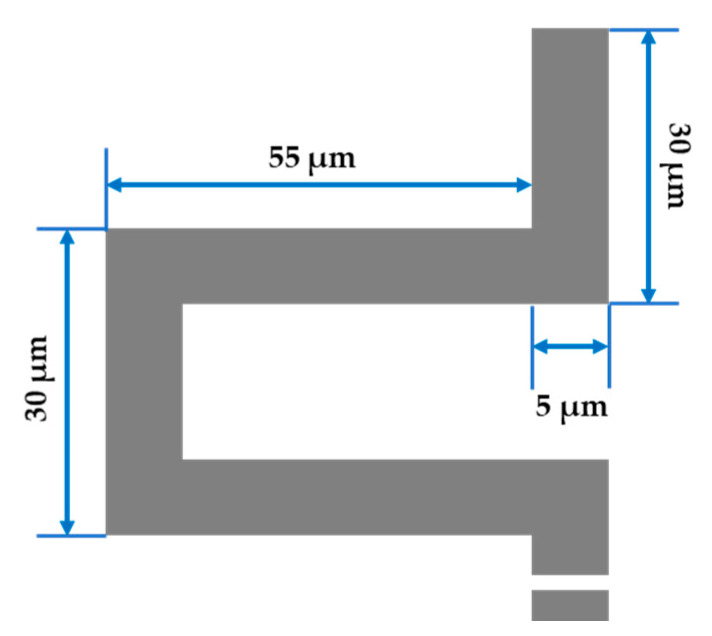
The element size for the coupled folded beam.

## Data Availability

Data are contained within the article. More detailed data and data presented in this study are available on request from the corresponding author. Part of them could be included in the Final reports to the corresponding funding organizations.

## Appendix A

In the following, expressions for variables in the analytical model are given. When the internal magnetic field $H_{eff}$ is uniform, the demagnetization matrix can be estimated by geometric parameters [43]. The effective demagnetizing factors $D_{ii}$ in the midplane of the rectangular magnetostrictive film are estimated by Equations (A1) and (A2)

(A1) $$D_{11}≅{\left(1+\frac{3}{4}\frac{L}{t_{m}}\left(1+\frac{t_{m}}{w}\right)\right)}^{-1},\;D_{22}≅{\left(1+\frac{3}{4}\frac{w}{t_{m}}\left(1+\frac{t_{m}}{L}\right)\right)}^{-1}$$

(A2) $$D_{33}=1-D_{11}-D_{22}$$

The magnetostrictive strain constitutive equation is given as Equation (A3):

(A3) $$\lambda_{m}=\left[\begin{array}{c} \lambda_{11}\\ \lambda_{12}\\ \begin{array}{c} \lambda_{13}\\ \lambda_{22}\\ \begin{array}{c} \lambda_{23}\\ \lambda_{33} \end{array} \end{array} \end{array}\right]=\left[\begin{array}{c} \lambda_{s}\left(M^{2}{}_{m1}-\frac{1}{2}(M^{2}{}_{m2}+M^{2}{}_{m3}\right))\\ \frac{3}{2}\lambda_{s}M_{m1}M_{m2}\\ \begin{array}{c} \frac{3}{2}\lambda_{s}M_{m1}M_{m3}\\ \lambda_{s}\left(M^{2}{}_{m2}-\frac{1}{2}(M^{2}{}_{m1}+M^{2}{}_{m3}\right))\\ \begin{array}{c} \frac{3}{2}\lambda_{s}M_{m2}M_{m3}\\ \lambda_{s}\left(M^{2}{}_{m3}-\frac{1}{2}(M^{2}{}_{m1}+M^{2}{}_{m2}\right)) \end{array} \end{array} \end{array}\right]$$

where $\lambda_{s}$ is the saturation magnetostriction, and $M_{mi}$ is the magnetizing component. Since the in-plane magnetization process is considered $M_{m3}=0$. According to the shear stress reciprocity criterion, the stress $\sigma_{m}$ of the magnetostrictive film under the static magnetic field H is expressed by Equation (A4):

(A4) $$\sigma_{m}=\left[\begin{array}{c} \sigma_{11}\\ \sigma_{12}\\ \begin{array}{c} \sigma_{13}\\ \sigma_{22}\\ \begin{array}{c} \sigma_{23}\\ \sigma_{33} \end{array} \end{array} \end{array}\right]=\left[\begin{array}{cccccc} C_{11} & C_{12} & C_{m,13} & 0 & 0 & C_{16}\\ C_{12} & C_{22} & C_{m,23} & 0 & 0 & C_{26}\\ C_{m,13} & C_{m,23} & C_{m,33} & 0 & 0 & 0\\ 0 & 0 & 0 & C_{44} & C_{45} & 0\\ 0 & 0 & 0 & C_{45} & C_{55} & 0\\ C_{16} & C_{26} & 0 & 0 & 0 & C_{66} \end{array}\right]\left[\begin{array}{c} \lambda_{11}\\ \lambda_{12}\\ \begin{array}{c} \lambda_{13}\\ \lambda_{22}\\ \begin{array}{c} \lambda_{23}\\ \lambda_{33} \end{array} \end{array} \end{array}\right]$$

From the calculated elastic matrix C, other structural properties, such as bulk modulus B, shear modulus G, and Young’s modulus $E_{m}$ of the magnetostrictive film can be derived using the Reuss–Hill approximation. The relationships between the upper and lower bound of bulk and shear modulus and elastic matrix components are defined as:

(A5) $$\begin{array}{c} B_{V}=\frac{C_{11}+C_{22}+C_{33}+2\left(C_{12}+C_{13}+C_{23}\right)}{9} \end{array}$$

(A6) $$\begin{array}{c} B_{R}=\frac{1}{\left(S_{11}+S_{22}+S_{33}\right)+2\left(S_{12}+S_{13}+S_{23}\right)} \end{array}$$

(A7) $$G_{V}=\frac{C_{11}+C_{22}+C_{33}-C_{12}-C_{13}-C_{23}+3\left(C_{44}+C_{55}+C_{66}\right)}{15}$$

(A8) $$G_{R}=\frac{15}{4\left(S_{11}+S_{22}+S_{33}\right)-4\left(S_{12}+S_{13}+S_{23}\right)+3\left(S_{44}+S_{55}+S_{66}\right)}$$

According to the expressions of bulk modulus $B=\frac{B_{V}+B_{R}}{2}$ and shear modulus $G=\frac{G_{V}+G_{R}}{2}$, Young’s modulus of the magnetostrictive film can be derived from $E_{m}=\frac{9BG}{3B+G}$.

## Appendix B

### Appendix B.1. The Geometric Parameters of The WCR

The WCR (Weakly coupled resonator) of the three different structures used in the main text are given as follows. The geometric parameters of the cantilever beam selected for the three basic structures are consistent, which are given in Table A1. The coupling beam structures used by the three structures are different and are given in Table A2. The position of the coupling beam is the distance from the fixed end of the cantilever beam. The size of the air domain is 10 × 10 × 10 mm.

**Table A1:** Geometric Parameters for the Cantilever Beam with Composite Films.

L	w	$t_{p}$	$t_{m}$	$t_{s}$
1.5 mm	0.1 mm	2 μm	2 μm	50 μm

**Table A2:** Geometric Parameters of the Coupled Beam for Different Structures.

Coupled Beam of WCR	Length	Width	Height	Couple Position
Structure 1	600 μm	5 μm	50 μm	350 μm
Structure 2	600 μm	5 μm	50 μm	300 μm
Structure 3	300 μm	10 μm	5 μm	300 μm

Where Structure 1 uses folded beams, the element size of which is given in Figure A1.

### Appendix B.2. The Material Properties of The WCR

The magnetostrictive material is based on Fe-based soft ferromagnetic materials, and the parameters are reasonably selected based on the current works. The key material parameters used by the analytical model and FEM are given as follows [30,56,62,63,64]:

The saturation magnetostriction $\lambda_{s}=130$ ppm, the saturation magnetization $M_{s}=\frac{0.8}{\mu_{0}}\;$, the effective magnetocrystalline anisotropy energy density $K_{u}=7.4$ KJ/m^3^, the in-plane easy-axis direction $\varphi_{EA}=\frac{\pi }{2}\;$, the rotational deviation $\delta_{EA}=0.6\%\;$ around the mean easy-axis angle $\varphi_{EA}$, the energy density deviation $\delta_{K_{u}}=15\%$ around the mean easy-axis angle $K_{u}$, and the static initial magnetic susceptibility $\chi_{m}=65$.

The AC voltage magnitude is $U_{0}=0.5$ V applied to the piezoelectric layer of R2, with the damping loss factor $\eta_{p}=\eta_{s}=0.001$, Young’s modulus of the magnetostrictive layer $E_{m}=100$ GPa, Poisson’s ratio of the magnetostrictive layer $\nu_{m}=0.27$, magnetostrictive material density $\rho_{m}=7870$ kg/m^3^, the piezoelectric layer $E_{p}=389$ GPa, $\rho_{p}=3300\;$ kg/m^3^, the substrate layer $E_{p}=160$ GPa, $\rho_{s}=2320$ kg/m^3^. The range of the values of the key performance-influenced parameters is given in the main text.
